# Microbial Metabolism in Soil at Subzero Temperatures: Adaptation Mechanisms Revealed by Position-Specific ^13^C Labeling

**DOI:** 10.3389/fmicb.2017.00946

**Published:** 2017-05-29

**Authors:** Ezekiel K. Bore, Carolin Apostel, Sara Halicki, Yakov Kuzyakov, Michaela A. Dippold

**Affiliations:** ^1^Department of Agricultural Soil Science, University of GöttingenGöttingen, Germany; ^2^Department of Soil Science of Temperate Ecosystems, University of GöttingenGöttingen, Germany; ^3^Institute of Environmental Sciences, Kazan Federal UniversityKazan, Russia

**Keywords:** psychrophiles, cryoprotectants, position-specific labeling, metabolic pathways, freeze tolerance

## Abstract

Although biogeochemical models designed to simulate carbon (C) and nitrogen (N) dynamics in high-latitude ecosystems incorporate extracellular parameters, molecular and biochemical adaptations of microorganisms to freezing remain unclear. This knowledge gap hampers estimations of the C balance and ecosystem feedback in high-latitude regions. To analyze microbial metabolism at subzero temperatures, soils were incubated with isotopomers of position-specifically ^13^C-labeled glucose at three temperatures: +5 (control), -5, and -20°C. ^13^C was quantified in CO_2_, bulk soil, microbial biomass, and dissolved organic carbon (DOC) after 1, 3, and 10 days and also after 30 days for samples at -20°C. Compared to +5°C, CO_2_ decreased 3- and 10-fold at -5 and -20°C, respectively. High ^13^C recovery in CO_2_ from the C-1 position indicates dominance of the pentose phosphate pathway at +5°C. In contrast, increased oxidation of the C-4 position at subzero temperatures implies a switch to glycolysis. A threefold higher ^13^C recovery in microbial biomass at -5 than +5°C points to synthesis of intracellular compounds such as glycerol and ethanol in response to freezing. Less than 0.4% of ^13^C was recovered in DOC after 1 day, demonstrating complete glucose uptake by microorganisms even at -20°C. Consequently, we attribute the fivefold higher extracellular ^13^C in soil than in microbial biomass to secreted antifreeze compounds. This suggests that with decreasing temperature, intracellular antifreeze protection is complemented by extracellular mechanisms to avoid cellular damage by crystallizing water. The knowledge of sustained metabolism at subzero temperatures will not only be useful for modeling global C dynamics in ecosystems with periodically or permanently frozen soils, but will also be important in understanding and controlling the adaptive mechanisms of food spoilage organisms.

## Introduction

Microbial processes in high-latitude ecosystems with permafrost or frozen soils during winter periods are important contributors to global carbon (C) and nitrogen (N) cycling. Metabolic activity has been detected in soil at temperatures as low as -39°C (Panikov et al., 2006; Schaefer and Jafarov, 2016) and an absence of subzero temperature limit for microbial metabolism was suggested by Price and Sowers (2004). Although biogeochemical models designed to simulate C and N dynamics in high-latitude ecosystems incorporate extracellular parameters (Schaefer and Jafarov, 2016), molecular and biochemical adaptations responsible for microbial freeze tolerance remain unclear. This knowledge gap hampers the estimation of C balances and ecosystem feedback responses. Consequently, tracing the metabolic pathways through which microorganisms transform organic substances under frozen conditions is crucial for unraveling the metabolic adaptation mechanisms (Scandellari et al., 2009; Dijkstra et al., 2011b; Dippold et al., 2014). Physiological studies have demonstrated that microorganisms remain metabolically active under frozen conditions, even at temperatures below -20°C (Panikov et al., 2006; Amato and Christner, 2009). A recent study has shown metabolism suggestive of microbial growth (DNA replication) at temperatures down -20°C (Tuorto et al., 2014). Therefore, a better understanding of how microorganisms circumvent challenges under frozen conditions is crucial. The challenges that organisms experience under frozen conditions include: (1) denaturation and loss of protein flexibility, (2) loss of membrane fluidity, which affects nutrient transport and decreases the activity of membrane-bound enzymes (Chattopadhyay, 2006), and (3) DNA and RNA secondary structures become more stable, inhibiting replication, transcription, and translation (D’Amico et al., 2006). Extracellularly, freezing leads to: (1) low water availability and activity (Oquist et al., 2009; Stevenson et al., 2015), (2) low thermal energy, slowing diffusion of nutrients, and excreted wastes to and from microorganisms (Hoehler and Jorgensen, 2013), and (3) high solute concentrations causing osmotic imbalance (Lorv et al., 2014). Intrusive ice crystals formed intracellularly and extracellularly can mechanically damage the cells (Gilichinsky et al., 2003). Several studies have been conducted to understand the survival strategies adopted by microorganisms to overcome these challenges. Attributes include the synthesis of cold-adapted enzymes that have high specific activities at low temperature (Herbert, 1989; Berry and Foegeding, 1997; Nakagawa et al., 2003), synthesis of antifreeze proteins (AFP) that bind to ice crystals, inhibiting their growth and recrystallization (Fletcher et al., 2001; Holt, 2003; Lorv et al., 2014), and adjustment of membrane composition by synthesis of unsaturated fatty acids to increase fluidity (Berry and Foegeding, 1997; Drotz et al., 2010).

Most of the studies on metabolism at low temperatures are based on isolated pure cultures from permafrost or used permafrost samples with inherently adapted microbes (Rivkina et al., 2000; Mykytczuk et al., 2013; Tuorto et al., 2014). However, subzero temperatures are also relevant to soils in temperate zones that freeze during winter. In frozen state, significant greenhouse gases are released from temperate soils, which make these soils a good analog for what we might expect from thawing permafrost (Nikrad et al., 2016). The importance of these soils in global C cycling while frozen and their vulnerability to thawing, calls for a better understanding of how microbes in these soils will contribute to global C feedback (Nikrad et al., 2016). This study was therefore designed to gain insights into microbial activities in temperate frozen soils. The study is bolstered by the novel method of applying position-specifically labeled substances as metabolic tracers in soil microbiomes (Scandellari et al., 2009; Dijkstra et al., 2011b; Apostel et al., 2015). Glucose has been identified as one of the most suitable candidates for tracing metabolic processes in soil, because it lacks physical and chemical interactions with mineral or organic soil components due to absence of charged functional groups or hydrophobic moieties (Fischer et al., 2010; Apostel et al., 2015). The use of position-specifically labeled glucose permits detailed reconstruction of microbial metabolic pathways and enables conclusions to be drawn on the microbial products formed under frozen conditions (Scandellari et al., 2009; Dijkstra et al., 2011a; Dippold and Kuzyakov, 2013; Apostel et al., 2015). This approach helps to identify the metabolic adaptations for overcoming the challenges under frozen conditions.

Analyses of ^13^C incorporated from specific glucose positions into soil, microbial biomass, dissolved organic carbon (DOC), and CO_2_ were conducted to identify microbial metabolic adaptations in frozen soil habitats. Poor water availability and low thermal energy limit microbial activity in frozen conditions. To survive these stresses, microorganisms form biofilms composed of exopolysaccharides (EPS). Sugars units forming the EPS are synthesized via the pentose phosphate pathway. Therefore, we hypothesized that the glucose C-1 position will be preferentially oxidized to meet energy demands. Consequently, high ^13^C recovery from the glucose C-1 position is expected in CO_2_. More of the remaining glucose C positions will be used for EPS synthesis than cellular compounds at subzero temperatures. This would result in higher ^13^C in the extracellular environment than in microbial biomass from the remaining glucose C positions due to the secreted EPS.

## Materials and Methods

### Sampling Site

The soils were collected (0–10 cm depth) from agriculturally used loamy Luvisol in northern Bavaria (49°54′ northern latitude; 11°08′ eastern longitude, 500 masl) in August 2014, sieved to 2 mm, and stored for 1 day at +5°C except for samples (*n* = 3) used to determine dry weight. Mean annual temperature and precipitation at the site are +7°C and 874 mm, respectively. The soil had a pH (KCl) of 4.88, a pH (H_2_O) of 6.49, a total organic carbon (TOC) and total nitrogen content of 1.77 and 0.19%, respectively, and a cation exchange capacity of 13 cmol_C_ kg^-1^.

### Experimental Design

Screw-cap glass microcosms (10 cm diameter and height of 12 cm) with a base layer of quartz sand were used for incubations. Eighty grams samples of soil were transferred to sample rings and installed on ceramic plates above the quartz sand. The soils were rewetted to field capacity by adding 10 ml of water to the underlying sand at +5°C for 2 days. Thereafter, the microcosms were preconditioned at +5, -5, and -20°C for 24 h. These temperatures were chosen to simulate (1) average annual soil temperature at the sampling site (control), (2) average winter temperature, and (3) lowest temperatures in some winters on this site and common deep freezer storage temperatures, respectively. Four position-specifically ^13^C-labeled isotopomers of glucose (^13^C-1, ^13^C-2, ^13^C-4, and ^13^C-6), uniformly ^13^C-labeled (U-^13^C) and unlabeled glucose (natural abundance background) were applied to the soils in separate microcosms with four replicates of each. 5 ml of 2.55 mM glucose solutions were applied on top of the soil. This C concentration was less than 5% of microbial biomass C and therefore, does not alter microbial community structure (Kuzyakov, 2010). Cups with 5 ml of 1 M NaOH were placed in each microcosm to trap CO_2_. 20% (w/v) of NaCl was dissolved in NaOH traps at -20°C to depress the freezing point. The microcosms were sealed and incubated at the respective temperatures. NaOH in the vials was exchanged after 10 h, 1, 2, 3, 6, and 10 days at +5 and -5°C and in addition after 15, 20, and 30 days at -20°C. Soil samples were collected after 1, 3, and 10 days at +5 and -5°C and also after 30 days at -20°C, to account for the slow processes expected in deeply frozen conditions. Thirty grams of each sample was immediately subjected to chloroform fumigation-extraction, as described below.

### Analytical Methods

#### Amount and δ^13^C Value of CO_2_

0.4 ml of each CO_2_ trap was diluted 1:10 with ultrapure water, and the CO_2_ content was determined with a non-dispersive infrared (NDIR) gas analyzer (TOC 5050, Shimadzu Corporation, Kyoto, Japan). The remainder of the CO_2_ in the NaOH traps was precipitated with 5 ml of 0.5 M SrCl_2_ solution. SrCO_3_ precipitates were separated by centrifuging four times at 2000 × *g* for 10 min and washing in between with Millipore water until pH 7 was attained. δ^13^C values of the dried SrCO_3_ (1–2 g) were measured with a Flash 2000 elemental analyzer coupled by a ConFlo III interface to a Delta V Advantage isotope ratio mass spectrometer (all Thermo Fisher Scientific, Bremen, Germany). ^13^C respired from the applied glucose was calculated according to a mixing model Eqs 1 and 2 (Gearing et al., 1991), where the C content of the background ([C]_BG_) in Eq. 1 was determined by Eq. 2.

(1) $$\begin{array}{c} {\left[C\right]}_{{\mathrm{CO}}_{2}}\;.\;r_{{\mathrm{CO}}_{2}}\;=\;{\left[C\right]}_{\mathrm{BG}}\;.\;r_{\mathrm{BG}}\;+\;{\left[C\right]}_{\mathrm{appG}}\;.\;r_{\mathrm{appG}} \end{array}$$

(2) $$\begin{array}{c} {\left[C\right]}_{{\mathrm{CO}}_{2}}\;=\;{\left[C\right]}_{\mathrm{BG}}\;+\;{\left[C\right]}_{\mathrm{appG}} \end{array}$$

where:

[C]_CO_2_/BG/appG_ C content of the sample/background/applied glucose (mg C g^-1^ soil)

r_CO_2_/BG/appG_^13^C atom %-excess of labeled sample/background/applied glucose (at %)

#### Quantification of Bulk Soil C Content and ^13^C Signature

Aliquots of soil were freeze dried, ground in a ball mill and 13–15 mg were weighed into tin capsules. ^13^C isotope measurements were performed with EuroVector elemental analyzers (HEKAtech GmbH, Wegberg, Germany) coupled by a ConFlo III interface to a Delta Plus XP IRMS (both units from Thermo Fisher Scientific, Bremen, Germany). C recovery from applied glucose was calculated according to Eqs 1 and 2.

#### ^13^C in Microbial Biomass and DOC Determination

Microbial biomass C was determined by chloroform fumigation-extraction. Two subsamples of 12 g were taken from each soil sample. One set of subsamples was extracted directly, while the other was first fumigated with chloroform for 3 days in a desiccator to lyse microbial cells. Thirty-six milliliters of 0.05 M K_2_SO_4_ was used to extract organic C on an orbital shaker for 1.5 h. Samples were centrifuged for 10 min at 2000 rpm and the supernatant was filtered for determination of C concentration (TOC/TIC analyzer, Multi C/N 2100, Analytik Jena, Jena, Germany). About 25 mg (fumigated) and 40 mg (unfumigated) freeze-dried extracts were used for δ^13^C determination via EA-IRMS, performed by the same instrument coupling used for bulk soil δ^13^C determination. Incorporation of glucose C into fumigated and unfumigated samples was calculated according to Eqs 1 and 2. Microbial biomass C and ^13^C were calculated by subtracting unfumigated from fumigated values and dividing by an extractability correction factor of 0.45 (Wu et al., 1990). C concentration and ^13^C recovery in unfumigated samples are DOC and its ^13^C content, respectively.

### Statistical Analysis

A Nalimov outlier test with 95% significance level was performed on CO_2_. ^13^C incorporation into bulk soil, microbial biomass, and CO_2_ were tested for significant differences between the positions, time of incubation and effect of temperature with a factorial analysis of variance (ANOVA). If assumptions of normality and homogeneity of variances with groups were not met, outcomes were validated by a non-parametric Kruskal–Wallis ANOVA. Significant differences were determined with Tukey’s honest significance difference (Tukey’s HSD) *post hoc* test at a confidence level of 95%. Statistical tests were performed with Statistica (version 12.0, Statsoft GmbH, Hamburg, Germany).

## Results

### CO_2_ and Recovery of Incorporated ^13^C

**FIGURE 1:**
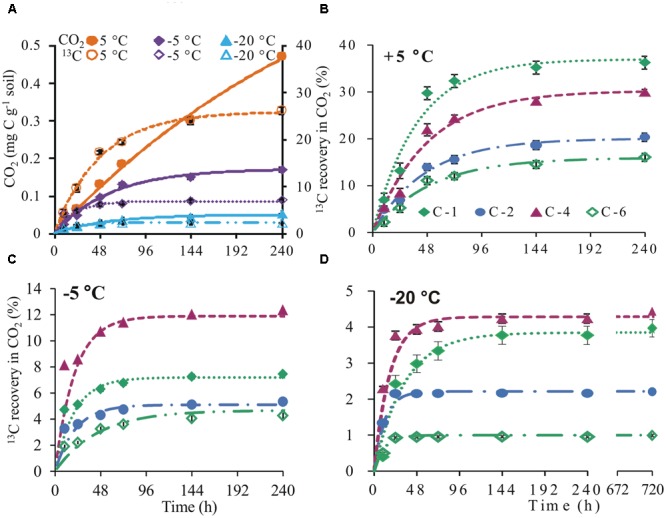
**Cumulative CO_2_ and ^13^C in CO_2_.** Cumulative CO_2_ (mean ± SEM, *n* = 4; solid symbols and continuous lines) and ^13^C (mean ± SEM, *n* = 4; open symbols and broken lines) recovered in CO_2_ released from uniformly labeled glucose **(A)** and cumulative ^13^C (mean ± SEM, *n* = 4) recovered in CO_2_ released from position-specifically labeled glucose at +5 **(B)**, –5 **(C)**, and –20°C **(D)**. ^13^C curves were fitted with non-linear least-square regressions according to an exponential equation [cum^13^C(*t*) = ^13^C_max_^∗^(1 – e^-^*^kt^*)], where cum ^13^C (*t*) is the cumulative ^13^C amount depending on time, ^13^C_max_ is the parametrically determined maximum of ^13^C, *k* is the mineralization rate, and *t* is time (parameter estimates in Supplementary Table S1). Steven’s runs test for the linearized, fitted ^13^C curves revealed no deviation from linearity (Supplementary Table S2). Significant differences (*p* < 0.05) between fitted curves are displayed in Supplementary Table S4.

CO_2_ was released down to -20°C, although cumulatively, there were 3- and 10-fold reductions at -5 and -20°C, respectively, compared to +5°C at day 10. Similarly, recovery of glucose-derived ^13^C in CO_2_ at -5 and -20°C was respectively 19 and 24% lower than +5°C (**Figure 1A**). At +5°C, ^13^C recovery in CO_2_ showed preferential oxidation of the glucose C-1 position. In contrast, the glucose C-4 position was the most respired at subzero temperatures. Irrespective of temperature, two phases of glucose C mineralization were observed: (1) high glucose ^13^C respiration within the first 3 days at 5°C and 2 days at -5 and -20°C and (2) low respiration of glucose-derived ^13^C thereafter (**Figures 1B–D**).

### ^13^C in Bulk Soil and Microbial Biomass

**FIGURE 2:**
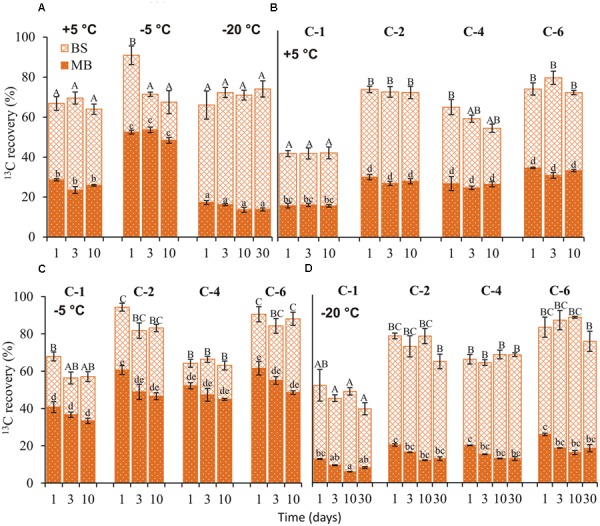
**^13^C recovery in bulk soil (BS) and microbial biomass (MB).** ^13^C recovery (mean ± SEM, *n* = 4) from uniformly **(A)** and position-specifically labeled glucose in BS and extractable MB, 1, 3, 10, and 30 (–20°C) days after application at +5 **(B)**, –5 **(C)**, and –20°C **(D)**. Total MBC at each temperature is provided in Supplementary Table S3. Significant effects (*p* < 0.05) of temperature, days, and individual glucose positions in BS are indicated by upper case letters above the error bars, and in extractable MB by lower case letters.

At day 1, ^13^C recovery in bulk soil from uniformly labeled glucose was over 24% higher at -5°C than at +5 and -20°C. After the first day, recovery did not differ with temperature (**Figure 2A**). Similarly, position-specific patterns of ^13^C recovery in bulk soil were comparable between +5 and -20°C but differed at -5°C. At +5 and -20°C, ^13^C recoveries in bulk soil from C-2, C-4, and C-6 were significantly higher (*p* < 0.05) than C-1 (**Figures 2B,D**). In contrast, recovery from C-2 and C-6 was significantly higher (*p* < 0.05) than from C-1 and C-4 at -5°C (**Figure 2C**).

From uniformly labeled glucose, the highest ^13^C recovery from microbial biomass was obtained at -5°C and it was two and three times higher than at +5 and -20°C, respectively (**Figure 2A**). The position-specific patterns of ^13^C incorporation into microbial biomass were not affected by temperature. The ^13^C recovery in microbial biomass from C-1 was ≈1.7 times lower than C-2, C-4, and C-6 at each temperature (**Figures 2B–D**). Moreover, recovery from each position did not differ over time at any incubation temperature.

The ratio of extracellular ^13^C recovery in bulk soil to microbial biomass was highest at -20°C (factor 3), followed by +5°C (factor 1.5) and -5°C (factor 0.6) (**Figure 2A**).

### Dissolved Organic Carbon

**FIGURE 3:**
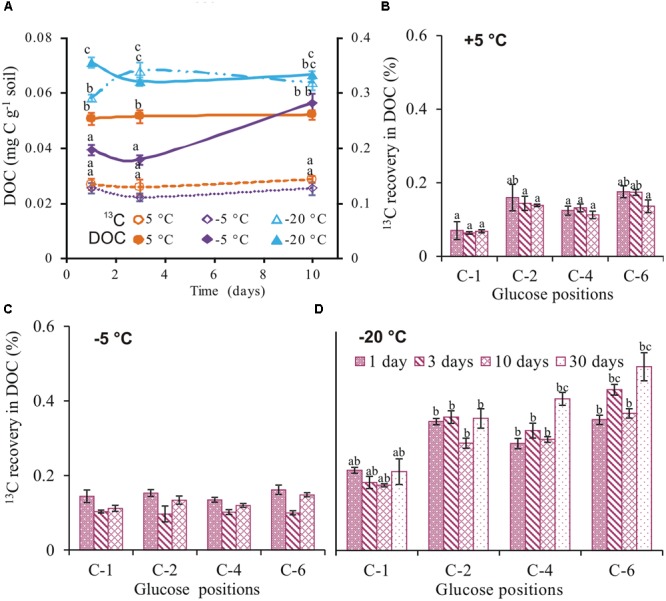
**DOC and ^13^C in DOC.** Total DOC (mean ± SEM, *n* = 4) extracted (solid symbols and continuous lines), ^13^C recovery (mean ± SEM, *n* = 4) from uniformly labeled (open symbols and broken lines) **(A)** and ^13^C recovery (mean ± SE, *n* = 4) from glucose positions in DOC at +5 **(B)**, –5 **(C)**, and –20°C **(D)**. Significant effects (*p* < 0.05) of temperature, days, and individual glucose positions in DOC are indicated by letters above the error bars.

Total extractable C in soil was highest at -20°C, being 1.4- and 2.3-fold higher than +5°C and -5°C, respectively (**Figure 3A**). Whereas DOC did not differ over time at -20 and +5°C, it increased by a factor of 1.5 between day 3 and 10 at -5°C, to reach a level similar to +5°C. ^13^C recovery in DOC at -20°C was also two times higher than both at -5 and +5°C (**Figure 3A**). Less than 0.4% of the applied ^13^C was recovered in DOC on day 1, irrespective of temperature (**Figure 3A**). The position-specific ^13^C patterns detected in CO_2_ were different to DOC at each temperature. Whereas recoveries in DOC at -5°C did not differ between the four glucose positions, the recovery from the glucose C-6 position was twice as high as C-1 at +5 and -20°C (**Figures 3B–D**).

## Discussion

### Metabolic Pathways Revealed by Glucose Mineralization

Microbial activity has been detected in soil down to -39°C (Panikov et al., 2006; Schaefer and Jafarov, 2016). This has evoked interest in the adaptive metabolic mechanisms of microorganisms at such extremely low temperatures. At subzero temperatures, low thermal energy is expected to slow down processes such as microbial substrate uptake (Hoehler and Jorgensen, 2013). However, less than 0.4% of applied ^13^C was recovered in DOC on the first day, irrespective of temperature (**Figure 3A**) suggesting that all applied glucose was taken up by microorganisms within the first day. These results agree with previous data showing that substrate uptake of highly available substances, such as glucose, is largely independent of temperature (Herbert and Bell, 1977; Schimel and Mikan, 2005). At the end of the incubation period, cumulative CO_2_ was 3- and 10-fold lower at -5 and -20°C than +5°C, respectively (**Figure 1A**). The results agree with studies showing strong temperature sensitivity of CO_2_ production in frozen soils (Monson et al., 2006; Panikov et al., 2006; Drotz et al., 2010). Irrespective of temperature, two phases of glucose C mineralization were observed: Phase 1 with high rates of ^13^C recovery in CO_2_ is consistent with intense glucose mineralization. Phase 2 thereafter, with low ^13^C recovery rates (**Figures 1B,D**), reflects mineralization of glucose-derived metabolites (Blagodatskaya et al., 2011). These results contradict those of Drotz et al. (2010), who observed low ^13^C in CO_2_ during phase 1 and high in phase 2. This discrepancy may reflect the soil types used for incubation. While our soils were agriculturally used loamy Luvisols, their soils were forest Podsols from the boreal region sampled from the organic horizon. Cold temperatures were shown to increase concentration of DOC in these soils (Haei et al., 2010), which implies a high concentration of low molecular weight organic substances and therefore low competition for applied substrate among microbes. In contrast, our agricultural soil was low in organic matter and the competition between microbes for applied glucose was highest immediately after application, leading to a fast uptake and release of glucose-derived CO_2_.

The ^13^C recovery pattern in CO_2_ showed high oxidation of the C-1 position in control soils (**Figure 1B**), and revealed that glucose was predominantly catabolized via the pentose phosphate pathway (Caspi et al., 2008; Dijkstra et al., 2011c; Apostel et al., 2015). Contrary to our hypothesis, metabolic behaviors completely switched over to a preferential respiration of the glucose C-4 position at -5°C and this behavior was still weakly visible at -20°C (**Figures 1C,D**). High ^13^C recovery from the C-4 position reflects glucose transformation via glycolysis (Dijkstra et al., 2011c; Apostel et al., 2013). Significant ^13^C recovery from the C-1 position, especially at -20°C, implies that, at such very low temperatures the pentose phosphate pathway again plays a greater role than at -5°C. This shift in metabolic pathways may reflect the need for antifreeze compounds. To confirm this interpretation, we assessed ^13^C incorporation into microbial biomass.

### ^13^C Incorporation into Microbial Biomass

Liquid water in soil is a prerequisite for microbial activity (Oquist et al., 2009). At +5°C, microbial activity was not constrained by water availability or slow substrate diffusion. The enzymes responsible for organic matter decomposition also remain active at this temperature (Razavi et al., 2015, 2016). Consequently, this temperature was favorable for glucose use, as reflected by the 4- and 13-fold higher ^13^C recovery in CO_2_ than at -5 and -20°C, respectively. Nonetheless, this high catabolic use of glucose-derived ^13^C with increasing temperature did not correspond to anabolic use. The highest ^13^C incorporation into microbial biomass was recorded at -5°C. The position-specific ^13^C recovery patterns in bulk soil and microbial biomass at +5°C were complementary to the metabolic fluxes observed in CO_2_, showing that glucose was predominantly metabolized via pentose phosphate pathway. The dominance of this pathway at similar temperatures under moderate C supply reflects the classical metabolic C allocation observed in previous studies (Dijkstra et al., 2011c). This is triggered by the need for pentose and NADPH for biosynthesis (Fuhrer and Sauer, 2009).

Although water availability, water activity, and thermal energy are low at subzero temperatures (Davidson and Janssens, 2006; Oquist et al., 2009; Stevenson et al., 2015), recent studies demonstrated microbial growth down to -15°C in permafrost isolates (Mykytczuk et al., 2013), -20°C in permafrost cores (Tuorto et al., 2014), and the microbial metabolism limit was set at -33°C (Rummel et al., 2014). Therefore, the substantial amount of ^13^C incorporated into microbial biomass we observed at -5°C is not surprising. To survive and grow at such extremely low temperatures, microorganisms induce a suite of physiological processes which include both metabolic and biomass adjustments (Wouters et al., 2000; Drotz et al., 2010). A better understanding of these metabolic and biomass adjustments will be necessary in estimating the role of microorganisms in biogeochemical cycles of frozen soils.

### Antifreeze Adaptation Mechanisms

**FIGURE 4:**
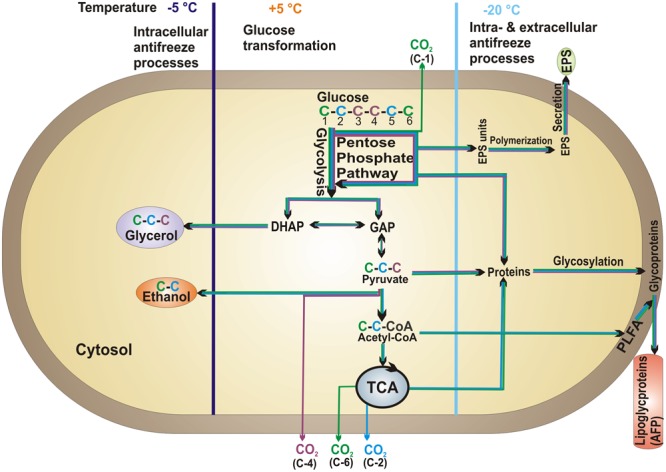
**Proposed model of microbial glucose transformation pathways at +5°C (central compartment) and antifreeze adaptation mechanisms at subzero temperatures (–5°C on the left and –20°C on the right compartments).** Colored arrows correspond to glucose C positions and indicate their fate.

^13^C incorporation into microbial biomass at -5°C was two and three times higher than at +5 and -20°C, respectively, and this incorporation made up more than two-thirds of the total glucose-derived ^13^C recovered in bulk soil (**Figure 2A**). In a recent study, ^13^C incorporation into microbial DNA was not detected after 1 month at subzero temperatures (Tuorto et al., 2014). This suggests that glucose utilization by microorganisms at -5°C were mainly intracellular metabolic responses to freezing and was rapidly activated. Intracellularly, for the vast majority of prokaryotic cells, contain no unbound water (Coombe, 2002). Hence, deleterious effects of freezing to these microbes, include, but are not limited to osmotic imbalance. Since water is not a limiting factor at -5°C, due to freezing point depression by increasing solute concentration in unfrozen volume (Brooks et al., 1997; Price, 2000), microorganisms invest energy in the synthesis of intracellular compounds that minimize effects of osmotic stress brought about by freeze-dehydration. This freeze-dehydration alters cell membrane ultrastructure and membrane bilayer fusion and causes organelle disruption (Mazur, 2004). Glycerol and ethanol were recently identified as abundantly synthesized compounds at -4°C (Drotz et al., 2010). The synthesis of these compounds is consistent with glucose transformation via glycolysis, which is confirmed by the high ^13^C recovery from C-4 in CO_2_. These compounds act as cryoprotectants and are compatible solutes against freeze-dehydration (Block, 2003). Glycerol also interacts with water through hydrogen bonds depressing its freezing point (Lovelock, 1954). Glycerol therefore limits intracellular damage caused by freezing, hence maintaining microbial activity and CO_2_ production at subzero temperatures (Drotz et al., 2010). We emphasize that conversion of glucose to alcohols (ethanol and glycerol) does not limit synthesis of other cellular compounds such as phospholipids and proteins. Nonetheless, conversion of glucose to alcohols explains the high glycolysis (C-4) and low pentose phosphate pathway (C-1) fingerprints (**Figure 1C**) observed at -5°C (**Figure 4**).

We obtained lowest ^13^C incorporation into microbial biomass at -20°C (1.3 and 3 times lower than at +5 and -5°C, respectively; **Figure 2A**. Nonetheless, the ratio of extracellular ^13^C to microbial biomass in soil was highest (ratio 5) at -20°C (**Figure 2A**). This high ratio could not be attributed to the non-metabolized glucose that remained in the soil because less than 0.4% of applied ^13^C was recovered in DOC at day 1 (**Figure 3A**); moreover, extracellular ^13^C recovery increased again at later time points. The high ^13^C recovery in bulk soil compared to microbial biomass at -20°C suggests that microorganisms utilized glucose for extracellular antifreeze compounds. To resist physical, environmental, and biological stress, and to survive in diverse ecological niches, microorganisms form biofilms. These biofilms are mainly composed of EPS secreted by microbes. Recently, culture-based studies revealed EPS secretion at -15°C by bacterial cells (Mykytczuk et al., 2013). In addition to cryoprotection, the high polyhydroxyl content of EPS lowers the freezing point and ice nucleation temperatures (Nichols et al., 2005; Qin et al., 2007; De Maayer et al., 2014). A key intermediate linking the anabolic pathway of EPS production and the catabolic pathway of glucose degradation is glucose-6-phosphate. In this step, the C flux bifurcates between the formation of glycolysis products and the biosynthesis of sugar monomers for EPS production via the pentose phosphate pathway (Welman and Maddox, 2003; **Figure 4**). Increasing glucose concentration was found to increase EPS production at low temperatures (Cayol et al., 2015). EPS production and secretion potentially explain the relevance of glycolysis and the pentose phosphate pathways, as reflected by preferential oxidation of glucose C-4 followed by C-1 positions (**Figure 1D**). This would account for the higher extracellular ^13^C in soil at -20°C compared to -5 and +5°C (**Figure 2A**). The poor extractability of EPS in water (Hughes, 1997) may explain why extracellular ^13^C was not entirely recovered in DOC at -20°C. Nevertheless, high ^13^C recovery in DOC coincided with high absolute DOC at -20°C (**Figure 3A**). This is consistent with the results of both Krembs et al. (2002), who found that EPS concentration in ice correlated positively with DOC, and with those of Hentschel et al. (2008), who observed the highest DOC content in forest soil at -13°C, followed by successively lower values at +5 and -3°C. This pattern, based on forest soils in a field experiment, agrees perfectly with our observation on agricultural soils in a laboratory experiment (**Figure 3A**), suggesting that underlying adaptation strategies are rather general. The spectroscopic properties of DOC at -13°C did not indicate lysis of microbial biomass induced by freezing (Hentschel et al., 2008), which confirm the maintenance of cellular integrity.

In addition to EPS, organisms commonly synthesize AFP to overcome freezing effects. These AFP have been identified in fungi, bacteria, plants, insects, and polar fishes (Holt, 2003). Some bacterial AFP are secreted as lipoglycoproteins or anchored on the outer cell membrane as lipoproteins, stabilizing membrane lipids at low temperatures (Kawahara, 2002). Based on adsorption inhibition, bound AFP inhibit rapid water movement between ice crystals, preventing destabilization of small ice crystal grains and thus minimizing ice recrystallization (Yu et al., 2010). For this reason, 3–8% of water remains unfrozen as thin films coating organo-mineral particles. This protects viable cells sorbed onto their surfaces from mechanical destruction due to intrusive ice crystals growing in frozen soil (Gilichinsky et al., 2003). The unfrozen water provides channels through which nutrients reach the cells and through which waste products are eliminated by diffusion (Rivkina et al., 2000). The synthesis of extracellular AFP further underlines the greater relevance of the pentose phosphate pathway (oxidation of C-1) at -20°C than at -5°C (**Figure 1D**). Increased pentose phosphate pathway activity signifies increased NADPH production, which is required as a source of reduction equivalents in protein and lipid synthesis (Nelson et al., 2008). Moreover, synthesis of hardly extractable extracellular AFP could also account for the fivefold higher extracellular ^13^C recovery in bulk soil than in microbial biomass at -20°C (**Figure 2A**).

The position-specific tracing of metabolic pathways in our study revealed that microorganisms induce a suite of temperature-dependent physiological processes to cope with freezing. This enables both catabolic and anabolic processes. These physiological changes reflect microbial resilience, enabling them to inhabit any ecological niche. Whether these responses are accompanied by a microbial community shift or solely reflect phenotypic plasticity remains to be determined.

## Conclusion

Position-specific ^13^C labeling proved to be a valuable tool in understanding the survival strategies of microorganisms at subzero temperatures. Glucose was metabolized by the pentose phosphate pathway at +5°C and this shifted to glycolysis at subzero temperatures. High ^13^C recovery within microbial biomass at -5°C suggests that microorganisms invest energy and resources in intracellular antifreeze metabolites. In contrast, strategies differ greatly at -20°C, where predominantly extracellular secretion protects the cells.

High-latitude C and N dynamic models such as Simple Biosphere/Carnegie-Ames-Stanford Approach (SiBCASA) focuses on extracellular parameters and only the thawing of permafrost is assumed to increase CO_2_ emission (Schaefer et al., 2011; Schaefer and Jafarov, 2016). This increase is likely to be overestimated because permafrost soils—even if completely frozen at low temperatures—exhibit considerable microbial activity. This newly gained information is useful in modeling C dynamics at high altitude and latitude regions with frozen soils, hence, improving the estimation of global C balances and ecosystem feedback responses.

## Author Contributions

The concept was contributed by YK and MD, experimental design by EB and MD, data acquisition and analysis by CA and SH, interpretation and drafting by EB, and revision including final approval of the version by YK and MD.

## Conflict of Interest Statement

The authors declare that the research was conducted in the absence of any commercial or financial relationships that could be construed as a potential conflict of interest.
