# Supplementary material for: Microbial Metabolism in Soil at Subzero Temperatures: Adaptation Mechanisms Revealed by Position-Specific 13C Labeling
Source: Front Microbiol. 2017 May 29;8:946. doi: 10.3389/fmicb.2017.00946 (PMC5447017; doi:10.3389/fmicb.2017.00946)
Supplement: Supplementary file 1 [file Table_1.DOCX]

**Supplementary Material**

**Original research**

Ezekiel K. Bore^*^, Carolin Apostel, Sara Halicki, Yakov Kuzyakov, Michaela A. Dippold

**Correspondence:** Ezekiel K. Bore, [ezekielbore7@gmail.com](mailto:ezekielbore7@gmail.com)

***Table S1.*** *Parameter estimates for the fitted ^13^C curves*

|  |  | 5 °C |  |  | -5 °C |  |  | -20 °C |  |
| --- | --- | --- | --- | --- | --- | --- | --- | --- | --- |
| Position |  | Estimate | p-value |  | Estimate | p-value |  | Estimate | p-value |
| C-1 | 13C(max) | 36.98275 | 0 |  | 7.18775 | 0.000000 |  | 3.843498 | 1.82E-12 |
|  | k | 0.025597 | 8.65E-09 |  | 0.04887 | 0.000000 |  | 0.029554 | 0.00093 |
| C-2 | 13C(max) | 20.05699 | 0 |  | 5.098329 | 0.000000 |  | 2.211274 | 0 |
|  | k | 0.02166 | 0 |  | 0.046749 | 0.000048 |  | 0.083188 | 2.43E-08 |
| C-4 | 13C(max) | 30.26421 | 0 |  | 11.89662 | 0.000000 |  | 4.28137 | 0 |
|  | k | 0.02094 | 0 |  | 0.04826 | 0.000000 |  | 0.05567 | 1.15E-09 |
| C-6 | 13C(max) | 15.95253 | 0 |  | 4.669367 | 0.000000 |  | 0.995224 | 0 |
|  | k | 0.01983 | 0 |  | 0.022271 | 0.000095 |  | 0.087748 | 3.78E-09 |

***Table S2.*** *Steven’s runs test for linearity: Regression analysis parameters for fitted ^13^C curves (ns= not significant)*

| Temperature | Position | R^2^ | P_slope≠0_ | P_runs test_ | P_equlity of slopes_ | P_equality of intercepts_ |
| --- | --- | --- | --- | --- | --- | --- |
| 5 °C | C-1 | 0.5353 | 0.0001 | 0.3000 | 0.3215 | 0.009379 |
|  | C-2 | 0.5980 | < 0.0001 | 0.3000 |  |  |
|  | C-4 | 0.8295 | < 0.0001 | 0.3000 |  |  |
|  | C-6 | 0.7541 | < 0.0001 | 0.3000 |  |  |
| -5 °C | C-1 | 0.9281 | 0.002 | 0.3000 | <0.0001 | ns |
|  | C-2 | 0.7506 | 0.0256 | 0.5000 |  |  |
|  | C-4 | 0.9801 | 0.0012 | 0.5000 |  |  |
|  | C-6 | 0.6170 | 0.0641 | 0.3000 |  |  |
| -20 °C | C-1 | 0.6316 | 0.0105 | 0.0714 | 0.2489 | <0.0001 |
|  | C-2 | 0.5287 | 0.0929 | 0.1071 |  |  |
|  | C-4 | 0.6674 | 0,0072 | 0.1071 |  |  |
|  | C-6 | 0.5134 | 0.117 | 0.0714 |  |  |

***Table S3.*** *Total microbial biomass C (mean ± SE) extracted during incubation*

|  | ∑MBC (µg g-1 dry soil) | |  |
| --- | --- | --- | --- |
| Temperature | 1 | 3 | 10 |
| 5 °C | 251.36 (6.47) | 199.31 (13.90) | 231.51 (7.89) |
| -5 °C | 300.76 (6.84) | 350.86 (8.04) | 237.41 (19.48) |
| -20 °C | 180.45 (10.58) | 193.98 (13.46) | 167.26 (7.92) |

***Table S4.*** *Significant differences (p<0.05) between the fitted curves for individual temperature are indicated by superscript letters on ^13^C (max) values.*

|  |  | Positions |  |  |
| --- | --- | --- | --- | --- |
| Temperature | C-1 | C-2 | C-4 | C-6 |
| 5 °C | 36.98^d^ | 20.06^b^ | 30.26^c^ | 15.95^a^ |
| -5 °C | 7.19^b^ | 5.1^a^ | 11.9^c^ | 4.65^a^ |
| -20 °C | 3.84 | 2.21 | 4.28 | 0.99 |
